# Draft Genome Sequence of a Novel *Serratia* sp. Strain with Antifungal Activity

**DOI:** 10.1128/MRA.01340-18

**Published:** 2018-12-06

**Authors:** Søs I. Dichmann, Byeonghyeok Park, Duleepa Pathiraja, In-Geol Choi, Peter Stougaard, Rosanna C. Hennessy

**Affiliations:** a Department of Plant and Environmental Sciences, University of Copenhagen, Frederiksberg C, Denmark; b Department of Biotechnology, College of Life Sciences and Biotechnology, Korea University, Seoul, South Korea; University of Maryland School of Medicine

## Abstract

This report describes the draft genome sequence of Serratia sp. strain S40, isolated from potato; it contains 5,383,735 bp and a G+C content of 55.9% and harbors 4,875 predicted coding sequences across 29 contigs. The genomic data provide insight into the genetics underpinning the antifungal activity of this strain.

## Abstract (precis)

This report describes the draft genome sequence of Serratia sp. strain S40, isolated from potato; it contains 5,383,735 bp and a G+C content of 55.9% and harbors 4,875 predicted coding sequences across 29 contigs.

## ANNOUNCEMENT

Serratia species are ubiquitous in the environment and have been isolated from soil, water, and animals, including humans (1). The genus comprises both nonpathogenic species and pathogens of plants, animals, and humans. Several members of the genus have been associated with the rhizosphere of diverse plants and shown to be plant growth promoting and antifungal (2–4). The application of rhizosphere biocontrol bacteria to control plant-pathogenic fungi is an alternative approach to the use of chemical fungicides.

Serratia sp. strain S40 was isolated from a potato rhizosphere in Zealand, Denmark (5). For whole-genome sequencing, the strain was cultivated in lysogeny broth (LB) for 24 h at 20°C before DNA extraction. Genomic DNA was purified using the Gentra Puregene Yeast/Bact. kit (Qiagen, Germany) according to the manufacturer’s instructions, and purified genomic DNA was verified by 16S RNA gene sequencing as previously described (5). Library preparation for whole-genome sequencing was performed using the Nextera DNA library prep kit (Illumina, Inc., San Diego, CA, USA). Sequencing was carried out with an Illumina MiSeq platform using MiSeq reagent kit V3 (600 cycles) (Illumina, Inc.). A total of 896,235 (539.5 Mbp) paired-end reads with a read size of 301 × 2 bp and average insert size of 478 bp were retrieved. Adapter sequences and low-quality bases (<Q20) were trimmed from raw reads using the TrimGalore! software version 0.4.4 (https://www.bioinformatics.babraham.ac.uk/projects/trim_galore/). A total of 885,343 reads (386.6 Mbp, 71.8× coverage for Serratia sp.) passed quality control and were used for assembly using SPAdes Genome Assembler version 3.7.1 (6). Genome annotation was conducted using P-CAPS (7) and the NCBI Prokaryotic Genome Annotation Pipeline (8).

The draft genome sequence of Serratia sp. S40 is 5,383,735 bp long, and its contigs are set up in 29 scaffolds (*L*_50_, 3; *N*_50_, 362,774 bp), with a mean G+C content of 55.9%. The annotation of the Serratia sp. S40 genome revealed the presence of 4,875 predicted coding sequences (CDS) and 93 predicted RNA (14 rRNAs, 79 tRNAs) coding sequences.

Using a series of *in silico* genome mining tools, a total of 8 secondary metabolite gene clusters were predicted by antiSMASH (9), including 4 nonribosomal peptide synthetase/polyketide synthase (NRPS/PKS) clusters; 3 bacteriocin gene clusters were identified by BAGEL3 (10), and NapDos (11) predicted 8 KS-domains and 10 C-domains, while NP Searcher (12) identified 1 NRPS gene cluster.

Further in-depth analysis of this genome will increase our understanding of the antifungal activity of this strain for applications within biocontrol and potentially lead to the discovery of new natural products.

### Data availability.

This whole-genome sequence project has been deposited at DDBJ/ENA/GenBank under the accession number QYYG00000000. The version described in this paper is QYYG01000000. Raw sequence files are deposited under the accession number PRJNA491277.
